# Two Cases of Hepatic Abscess, Treated in the Mercy Hospital by W. H. Byford, M. D.

**Published:** 1858-09

**Authors:** E. O. F. Roler

**Affiliations:** Resident Physician


ARTICLE III.

### TWO CASES OF HEPATIC ABSCESS, TREATED IN THE MERCY HOSPITAL BY W. H. BYFORD, M.D.

REPORTED BY E. O. F. ROLER, RESIDENT PHYSICIAN.

Michael F——, aged twenty-four, a native of Louxemburg, Germany, a tailor by trade and of temperate habits, admitted into the medical wards of Mercy Hospital, April 12th, 1858. Has never been of a robust constitution, and has a strumous appearance; but, with the exception of one or two attacks of ague, has always been exempt from any special disease.

*History.*—The first symptoms of his present illness made their appearance about ten weeks previous to his admission. A dull, aching pain was felt in the right hypochondrium, which rapidly increased in intensity, and was accompanied with febrile action of a high grade, pain in the right and left shoulder, hurried breathing, short, dry cough, inability to lie on either side, with nausea and frequent efforts at vomiting. Says his alvine evacuations have been of a "whitish color" from the beginning of his disorder. The general symptoms subsided after eight or ten days, but the pain and sense of tension in the right side continued to harrass and weaken the patient up to the time of admission.

*Present Appearance and Symptoms.*—Patient considerably emaciated; countenance dull and apathetic; skin and conjunctiva of eyes of a deeply jaundiced hue; tongue moist, and coated with a dark yellow fur; pulse small, compressible and frequent; bowels torpid, moving only when acted upon by purgatives. On examination, a well defined tumor is found to exist in the right hypochondrium, extending across the epigastrium and into the umbilical and right lumbar regions. An obscure outline of the right lobe of the liver can be traced along its boundary in the abdomen, and the whole surface seems slightly indurated and exceedingly tender on pressure. The tumor, externally, has a tense and glistening appearance, but there is no discoloration of the surface nor any of the characteristic signs of inflammation in the underlying textures. No fluctuation perceptible; the extreme tenderness, however, prevents a satisfactory examination. Patient thinks he has had nothing like rigors, but has sweat freely at times during the last week or ten days, with evening exacerbations of fever.

*Diagnosis.*—Acute inflammation of the liver, resulting in suppurative abscess.

*Treatment.*—A poultice of ground flaxseed applied over the swelling; internally, hyd. sub. mur. gr. iij., divided into four powders, and given every four hours until the whole are taken; afterwards mag. sulph, q. s. to move bowels freely.

*April 13th.* Had two copious clay-colored evacuations during the night; restless and complains of inability to sleep, and a sensation of *tearing* when attempting to lie on either side. Appetite greatly impaired; thirst moderate; pulse this morning 104; has been sweating freely since three o'clock a. m. Ordered tr. cinch. ʒj. every six hours, to which add, at the time of taking, ac. sulph. ar. gtt. xv., and an opiate at night to procure rest.

*April 14th.* Late last night patient began to complain of sharp pains over the whole abdominal region; his pulse became thread-like and 148 per minute. He described them as "cutting pains," and most severe in the vicinity of the tumor, and radiating from it as a centre. Fomentations were applied, and morph. gr. $\frac{1}{3}$ was given at intervals, until the severity of the symptoms subsided.

11 *a. m.* Is yet suffering some pain; abdomen swollen, tense and painful on slightest pressure; pulse 124; breathing 35; position dorsal, knees drawn up.

Dr. B. remarked to the class present that the symptoms evidently indicated a change in the condition of the patient since the last examination. There are three different ways in which an hepatic abscess may terminate, all of which *usually* prove fatal.

1*st.* From contiguity of surfaces, there may be an inflammatory adhesion between the peritoneal coverings of the liver and abdomen, and the contents of the abscess find an exit externally.

2*d.* By the same process, it may find its way into some of the thoracic or abdominal viscera, as the lungs, stomach and transverse colon.

3*d*. There may be an effusion into some of the serous cavities, as the pleura, the pericardium, or more frequently the peritoneum.

In the majority of cases, the first and second modes of termination prove fatal, and in the event of the last taking place, death would almost inevitably follow. Had any one of these results transpired in the case before us? To a certain extent the diagnosis was easy. Peritoneal inflammation of a moderately severe grade evidently existed, but was it of sufficient intensity to demonstrate the presence of an irritating fluid of any kind in the abdominal cavity? He remarked that an important fact was to be remembered during the suppurative process of an abscess, especially of that character. The breaking down of the textures and their solution were always preceded by a certain degree of inflammatory action, forming the *adhesive* stage in its progress through the part. Contiguous parts and organs would thus become agglutinated during this transition stage, and immediate effusion prevented. When this inflammatory action involved serous structures, the constitutional and local symptoms were always more or less aggravated. Might it not be of that character in the case before them? Might not an inflammatory adhesive action be going on between the peritoneal covering of liver and abdomen, and a consequent increase in the intensity of the symptoms? He was inclined to this opinion from two circumstances.

1*st*. There was no perceptible subsidence of the tumor, which ought to take place in the event of a discharge of the pus into the peritoneal cavity.

2*d*. The symptoms were not of as severe a grade as should be expected, and which would most likely occur under those circumstances.

Yet no positive diagnosis could be given then as to which of the two conditions did exist. In a very short time, however, there would be satisfactory evidences.

The previous treatment to be continued, with the addition of morph. gr. ¼ at sufficient intervals to keep the patient confortable.

*April* 15*th*. 8 *a. m.* Pulse 130; abdomen quite tympanitic, but not painful; has been sweating profusely; bowels constipated.

Ordered ol. ric. ℥vj., ol. tereb. gtta. xx. After bowels have moved, the tonic mixture to be given as before; also, pot. nit. gr. x., morph. sul. gr. ¼, every four hours.

9 *p. m.* Had a copious clay-colored evacuation during the day. Pulse 149; breathing 38; restless.

*April* 16*th*. 9 *p. m.* Pulse 158; breathing 40; tumor has increased in size; in great distress from distension and pain in the side. Treatment continued.

*April* 17*th*. Complains of severe pleuritic pains above the swelling over the whole right side of the chest.

On careful examination, a deep, obscure fluctuation can be detected in the prominent part of the tumor. As the symptoms were rapidly becoming more serious, from an extension of inflammatory action, and Dr. B. believing that a sufficient amount of adhesion had already taken place between the surface of the liver and the abdominal parietis, he determined to give it vent externally without further delay. A small trocar was thrust in at the point of fluctuation to the depth of a couple of inches, and a narrow opening made in the direction of the obliq. ext. muscle. Its withdrawal was followed by a rush of gas, intolerably fetid, and a quantity of dark, sero-purulent fluid, with which were intermingled minute, cheesy-looking particles and shreds, which, under the microscope, were found to be of a tuberculous character. About a pint and a half was discharged with a considerable lessening of the distension, to the great relief of the patient. A tent was then introduced, and a poultice of flaxseed applied over the whole surface. Ordered tr. cinch. ℥j., quin. sulph. gr. j., with ar. sul. ac. gtt. xv. every six hours.

*April* 18*th*. Became restless during the night, with symptoms of sinking. Gave brandy punch every hour or two until pulse came up.

9 *p. m.* Pulse 108. No discharge since yesterday; distension again taking place. Introduced a probe, which was followed by an emission of fetid gas, with the escape of six or seven ounces of dark serous fluid, and a small quantity of healthy-looking pus.

*April 19th. 9 p. m.* Pulse 100. A small quantity of serous pus flows from the opening continually. Tonic treatment continued.

*April 20th.* The discharge being interrupted from a constant clogging up of the small aperture within, the knife was inserted the second time and the opening enlarged, which resulted in the immediate evacuation of a pint of thick, offensive pus.

*May 11th.* The patient has been kept on the same treatment since last date. A slight discharge from the abscess has taken place daily with occasional probing, but is gradually decreasing. Patient has improved in strength and appearance, sitting up several hours during the day, with moderately good appetite. His bowels have been torpid, but readily acted upon by mild purgatives, especially the salines. Dejections have all been of a light clay color. Quinine discontinued, and tr. cinch. with ar. sul. ac. given as before.

*May 13th.* Early this morning, while getting out of bed, the patient suddenly felt an increased flow from his side, and, on removing the cloths, found them saturated with a fluid of an intensely yellow color. On examination, it was found to possess an alkaline reaction, and to have all the properties of hepatic secretion, and oozing out continuously with the purulent matter from the opening.

Dr. B. remarked, upon his next visit, that the gall bladder had evidently given way under an extension of ulceration, as so large a discharge could not otherwise be accounted for.

*May 14th.* Had an evacuation of natural consistence, but of a chalky whiteness. Bile continues to pass out freely with the ordinary matters, which have almost entirely ceased.

*May 16th.* Discharge of bile continues.

Ordered a piece of oil silk to be laid over the fistula and compress applied, with a view of directing it again in its proper channel.

*May 17th.* Had a chill last evening, which lasted half an hour, and was followed with slight febrile reaction. Compress removed, and an ounce of viscid bile and pus escaped. Compress again applied. Bowels constipated. Ol. ric. ℥j. Afterward tonic treatment, as before.

*May 21st.* Complains of headache, want of appetite, and has become intensely icteric. Compress removed daily, and, on slight pressure, a quantity of bile and pus escapes—about equal parts of each—and occasionally a small quantity of transparent ropy fluid. Ordered quin. sulph. gr. xv., divided into four parts, and given at intervals during next twenty-four hours.

*June 2d.* The icteric hue, which had almost entirely disappeared, has returned with greater intensity than before. A deep yellow coat on the tongue, and the patient's saliva, which is secreted abundantly, has a perceptible yellow tinge. Bile is also detected in his urine. Fistula discharging pus, but no bile.

*June 3d.* Last night, was seized suddenly with an excruciating pain in the right hypochondrium, extending into the epigastrium, and a heavy pain also in the lumbar region. His pulse became slightly increased in rapidity, but compressible, and the skin moist and not above the normal temperature. The distress was evidently caused by the passage of a calculus along the biliary duct. Patient continued to suffer exceedingly for a couple of hours, with knees flexed on the abdomen, and able to lie only on the right side. Applied fomentations of hops, and gave morph. gr. $\frac{1}{4}$ at short intervals, until he became quiet. Ordered this morning,

| | |
|---|---|
| Hyd. sub. mur., | gr. iv. |
| Sod. bi-carb., | ℈ij. |
| Morph. sulph., | gr. j. |

Divided into four powders, and give one every three hours; afterward, the tonic mixture as before.

*June 9th.* Pain in side; anorexia and restlessness. There has been no discharge of bile for several days, but an increase of healthy pus, and a constant flow of the muco-watery fluid spoken of previously. Yellow hue of the skin continues. Probed the fistula thoroughly, to induce a reflow of the biliary secretion, which has evidently been obstructed in its passage through the fistula, and is the consequent cause of an aggravation of symptoms. Prescribed nit. ac. ʒj., aqua ℥ij. Give ʒj. every six hours, instead of tr. cinch. and elix. vit.

*June* 10*th*. Bile again flowing out. Patient slightly better.

*June* 15*th*. Had an evacuation this morning of nearly natural appearance. The icteric hue is disappearing, and the patient seems to be gradually improving in every respect.

*June* 25*th*. Is able to walk out of doors, but complains of a dragging sensation in the right side, which is relieved by wearing a supporting band. Stools are slightly bilious, but not entirely natural. But little discharge of any kind.

*July* 2*d*. Feels well. Has concluded to go home, but will continue to use the nitric acid mixture for a couple of weeks yet.

*Aug*. 3*d*. Called at the office to-day in fine health and spirits. The fistula has closed up entirely, and there is no pain or tenderness on moderate pressure. Says he feels as if something bound him in the side when standing erect and throwing his head and shoulders backward; otherwise, he feels as well as he has ever been.

During the time the patient was icteric, or the bile flowed through the fistula freely, no evidence of bile existing in the fæces could be detected upon common examination. From the time it began to greatly diminish in flow, the patient was jaundiced, and he suffered under the poisoning influence of bile in the circulation; this latter subsiding gradually, as yellow fæces began to make their appearance.

Case 2. J. T., about thirty years of age, an Irish laborer, of dissipated habits, admitted July 9th, 1858. Had generally enjoyed good health, until within the last year his constitution had become weakened from exposure and excessive use of alcoholic drinks.

*History*.—Three months previous to his admission, he began to complain of a dull, heavy pain in the right hypochondriac region, accompanied with loss of appetite and impaired digestion, which, in a short time, debilitated him to such a degree, that he was compelled to abandon his daily labor. A gradual accession of other morbid symptoms succeeded, and, at the end of eight weeks, he was unable to leave his bed. At about this stage of the disease, an indurated swelling was observed at the seat of pain in his side, which continued to enlarge, and, at the time of admission, the painful distention seemed to constitute the chief distress of the patient. Has had but little medical treatment, and been but indifferently supplied with the means requisite to comfort, being at one time an inmate of the county house.

His condition at time of admission is one of extreme emaciation. Eyes are sunken; upper lip retracted, and countenance expressive of the most intense suffering. Tongue coated with a brown fur; breathing rapid; pulse feeble and frequent; skin sallow, and bathed in a profuse cold sweat. Is able to lie only on the right side with knees flexed, and is tormented with a constant vomiting of dark bilious matters. Bowels obstinately constipated. A large fluctuating tumor distends the epigastrium and right hypochondrium, and encroaches on the diaphragm, preventing its descent during inspiration. On applying the ear, a well marked friction sound is audible over the whole surface, showing an absence of adhesion between it and the abdominal parietis.

The patient's greatest distress arises from a constant dragging down, and a sensation as if some of the membranes or viscera were being torn from their attachments within the abdomen.

*Diagnosis.*—Inflammatory enlargement of the liver, with extensive abscess. The prognosis being hopelessly unfavorable, little else than palliative treatment was deemed advisable. A poultice, wet up with an infusion of aconite, was placed over the tumefaction; internally, hyd. sub. mur. gr. ij., rhei. pulv. gr. xv., every two hours until the bowels move.

*July* 10*th*. Much the same; had an evacuation of hard, brown-colored fæces.

Prescribed morph. gr. $\frac{1}{4}$, every three hours; also, pot. iod. ʒj. aqua ℥ij., dose ʒj. every four hours.

9 *p. m.* More comfortable and dozing.

*July* 11*th*. About midnight was seized with lancinating pains over the whole abdomen, which became tympanitic and exceedingly tender to the touch. Patient has continued in extreme agony, notwithstanding large doses of opiates internally and anodyne fomentations locally; pulse almost imperceptible; giving brandy with morph., frequently repeated.

*July 12th.* Died at three o'clock this morning.

*Autopsy, ten hours after death.*—The peritoneal membrane showed evidences of intense inflammation, and its cavity was filled to the utmost tension with effused fluid, with which were intermingled a quantity of dark purulent matters and shreds of coagulated lymph. The liver was the only organ examined. The left lobe was slightly larger than normal, very firm and elastic to the touch. The gall bladder contained an ounce of greenish viscid bile. The right lobe was enormously enlarged, occupying the whole right hypochondriac, the epigastric, umbilical and right lumbar regions, and pressing upwards on the diaphragm. Slight adhesions had taken place along the outer border of the lobe, and at a little to the right of the centre of the convex surface—which was a mere film in depth—a small opening existed, about the size of a large crow quill, leading to an extensive abscess, containing about three pints of dark, thick, fetid pus. Through this opening the contents had found an exit into the peritoneal cavity, and produced death from the inflammation and effusion consequent upon its irritant action.

---

ARTICLE IV.

